# Use of a Dense Single Nucleotide Polymorphism Map for In Silico Mapping in the Mouse

**DOI:** 10.1371/journal.pbio.0020393

**Published:** 2004-11-09

**Authors:** Mathew T Pletcher, Philip McClurg, Serge Batalov, Andrew I Su, S. Whitney Barnes, Erica Lagler, Ron Korstanje, Xiaosong Wang, Deborah Nusskern, Molly A Bogue, Richard J Mural, Beverly Paigen, Tim Wiltshire

**Affiliations:** **1**Genomics Institute of the Novartis Research Foundation, San DiegoCaliforniaUnited States of America; **2**The Scripps Research Institute, San DiegoCaliforniaUnited States of America; **3**The Jackson Laboratory, Bar HarborMaineUnited States of America; **4**Celera Genomics, RockvilleMarylandUnited States of America

## Abstract

Rapid expansion of available data, both phenotypic and genotypic, for multiple strains of mice has enabled the development of new methods to interrogate the mouse genome for functional genetic perturbations. In silico mapping provides an expedient way to associate the natural diversity of phenotypic traits with ancestrally inherited polymorphisms for the purpose of dissecting genetic traits. In mouse, the current single nucleotide polymorphism (SNP) data have lacked the density across the genome and coverage of enough strains to properly achieve this goal. To remedy this, 470,407 allele calls were produced for 10,990 evenly spaced SNP loci across 48 inbred mouse strains. Use of the SNP set with statistical models that considered unique patterns within blocks of three SNPs as an inferred haplotype could successfully map known single gene traits and a cloned quantitative trait gene. Application of this method to high-density lipoprotein and gallstone phenotypes reproduced previously characterized quantitative trait loci (QTL). The inferred haplotype data also facilitates the refinement of QTL regions such that candidate genes can be more easily identified and characterized as shown for *adenylate cyclase 7.*

## Abstract 2

A large resource of genetic markers, typed in 48 strains of mice, combined with statistical techniques, allow "in silico" mapping of genetic regions involved in interesting traits in mice

## Introduction

The combined efforts of the public and private mouse genome sequencing consortiums have yielded important advances in understanding the structure and content of the genome (Mural et al. 2002; Waterston et al. 2002). Identification of new genes from the sequence data and placement of all genes, along with genetic markers, on a physical assembly has greatly aided in the search for phenotypically important genes in both quantitative trait loci (QTL) and mutagenesis-based mapping. The sequencing of four different strains of laboratory mice for the initial genome assemblies also produced a catalog of natural sequence variations that are present between these commonly used strains. Other smaller scale resequencing efforts have increased the breadth of this information by including additional strains (Lindblad-Toh et al. 2000; Grupe et al. 2001; Wade et al. 2002; Wiltshire et al. 2003).

The utility of this sequence-variation data is 2-fold. First, the single nucleotide polymorphisms (SNPs) identified by these sequencing projects provide denser coverage marker sets that are well suited for high-throughput genotyping systems. Currently, these benefits have only been available for crosses between the relatively few strains for which substantial polymorphism discovery has been undertaken.

Second, the distribution of SNPs between any two strains, or more precisely, the lack of SNPs between two mouse strains, indicates regions of their genomes that were inherited from a common ancestor. Phenotypic differences that are traditionally mapped in QTL studies are almost exclusively due to loci inherited from different ancestral progenitors rather than new mutations (Frazer et al. 2004). Thus, a detailed knowledge of where common ancestral regions lie between strain pairs would speed QTL mapping by elimination of shared regions from consideration as candidate loci (Wade et al. 2002). Additionally, it has been proposed that the actual haplotype structures marking these ancestral relationships can be determined and that the relationship of haplotype distribution among mouse strains and phenotypic variation could be used to directly map the genetic controls for the phenotypes (Grupe et al. 2001). However, three major factors have seriously curtailed the implementation of in silico mapping methods: a lack of the necessary SNP density and distribution along the genome for more than just a few strains; incomplete phenotype data for multiple strains, and lastly, the appropriate analysis tools for making genotype–phenotype associations. More recently uncertainties have also been expressed concerning the level of data that will be required to make in silico mapping a viable method. This is in part due to the emerging complexity of the haplotype structure in mouse and also to such issues as how many strains need to be phenotyped to be able to gain statistical power for in silico mapping (Darvasi 2001; Frazer et al. 2004; Yalcin et al. 2004).

To overcome the barriers to in silico mapping, 10,990 SNP assays have been typed against 48 mouse strains in this study. These assays provide an extensive polymorphic marker set enabling expansion of traditional mapping efforts to other strains. Wide-ranging phenotyping projects that have been coordinated by The Jackson Laboratory (http://www.jax.org/phenome) have collated multistrain phenotype data. We demonstrate that when using these datasets, in combination with new analysis methods, statistically significant associations between discrete genomic regions and biologically important phenotypes can be identified. Confirmation of these associations was obtained by comparison to data from traditional QTL mapping methods.

## Results

SNP assays were designed based on sequence data from the Celera Mouse SNP Database and typed, in duplicate, against the genomic DNA of 48 strains of mice, including all 40 Mouse Phenome Project priority strains (see list of strains in Tables S1 and S2) (Bogue 2003). Twelve wild-derived inbred strains were included in the set. Two strains, SPRET/EiJ and SEG/Pas *(Mus spretus),* represent a different species of mouse from the other lines tested. Not surprisingly, fewer genotypes were obtained from these two because of the divergent genomes (Tables S1 and S2) of the distinct species, which led to a higher failure rate in the genotyping reactions. For the 36 non-wild-derived strains typed, 8,349 SNP assays produced at least 90% of the possible allelic data.

**Figure 1:**
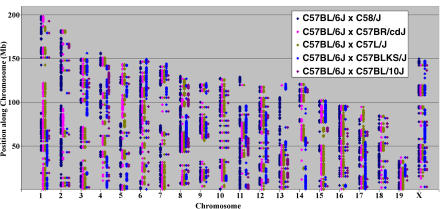
Visualization of the SNP Sets Allows for Mapping in Crosses That Minimize the Number of Potential Modifiers When the distribution of the SNPs is plotted out genome-wide, the expected irregular clustering of SNPs mark regions where heterozygosity was continuing to segregate during the inbreeding of the C57 family. Likewise, there are regions that were successfully homozygosed before the split of C58/J from the rest of the family members. In all five strain comparisons, no SNPs were found in the distal 25 Mb of MMU19.

Previously, sufficient polymorphic markers have not been available for many strain–pair combinations. The development of this SNP panel provides a resource of polymorphic markers to enable traditional mapping projects between almost any strain–pair combination of the 48 strains. In mapping a phenotype, introduction of modifier genes can be a confounding influence, and selection of more closely related mapping partners can alleviate this problem. The SNP density in this set is sufficient that mapping can now be accomplished between strains that had previously been too closely related for sufficient markers to be found. For example, for C58L/J by C57BL/6J and C57BL/6J by C57BL/10J comparisons, over 2,000 and 400 polymorphic markers, respectively, are available. Although large gaps do exist in the coverage of these strain–pair combinations, markers are present on all chromosomes to allow for initial candidate region identification (Figure 1). Details of all allele calls and SNP assays are available in Dataset S1.

**Figure 2:**
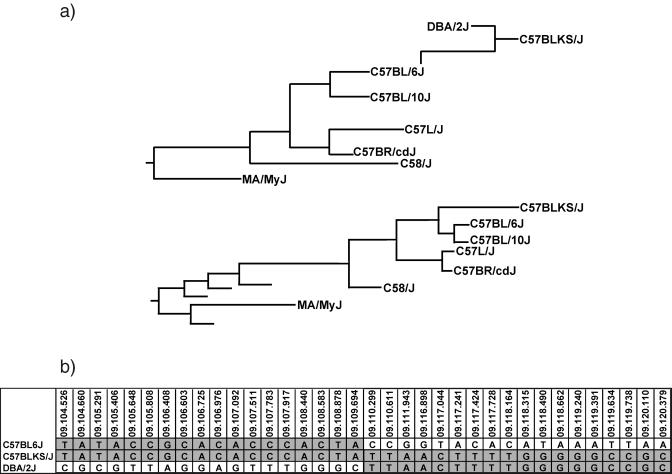
Genome-Wide SNP Data Accurately Represent the Known Ancestries of the Genotyped Strains (A) A tree, adapted from Beck et al. (2000), tracing the lineage of the C57 family of mice (upper tree) shows almost perfect correlation with a phylogenic tree based solely on SNP data (lower tree). The only difference in the two trees is the location of C57BLKS/J, which splits from C57BL/6J sooner in the phylogenic tree because of the genomic contributions of the non-C57 strain, DBA/2J. The maximum parsimony phylogenic tree of the strain relatedness was built using the pseudoalignment of the 10,990 SNP alleles for 48 strains with the Phylip package, version 3.6b. (B) The DBA/2J contribution to C57BLKS/J can be visualized in its allelic patterns. The region from 104 Mb to 109 Mb on MMU9 shows the same SNP alleles for both C57BLKS/J and its other parental strain, C57BL/6J (a period represents identity with the C57BLKS/J allele). At 110 Mb, the pattern switches and every C57BLKS/J allele matches the DBA/2J content through 120 Mb. SNP marker names are positioned above the alleles with the first number representing the chromosome the marker is located on, the second number being the Mb position on the chromosome, and the third number being an approximate location within the Mb.

Just as in humans, a spectrum of phenotypic values can be observed among the inbred strains of mice. SNPs that occur between these strains may produce a specific functional change in a gene leading to this phenotypic variation but are more often simply markers for an ancestral haplotype. The goal of in silico mapping is to identify which haplotype patterns (genetic measure) track with the phenotypic outcome with the idea that these haplotypes contain causative mutations. For in silico mapping to be successful, a requirement is that the SNP data accurately represent this ancestral relationship of the mice at the genomic level. At a gross level, this was examined by comparing branches of the phylogenic tree generated from this SNP dataset with the known breeding histories of the strains used in this study (Beck et al. 2000). An inspection of the C57-related family of mice, derived from a tree built from the SNP data of all 48 strains (Figure S1), recapitulates the family's lineage in the phylogenic tree (Figure 2A).

At a more detailed level the specific genomic contributions of mouse strains derived as hybrids of other common laboratory strains can be estimated. For example, DBA/2J is considered to have contributed approximately 16% of the genomic content to the C57BLKS/J mouse (Naggert et al. 1995). Comparisons of C57BL/6J, the other founder strain of C57BLKS/J, and DBA/2J-specific alleles to the SNP content of C57BLKS/J clearly defines these large regions of DBA/2J contribution as shown for Mus musculus Chromosome 9 (MMU9) (Figure 2B). Based on the SNP data, it can be estimated that 20% of the C57BLKS/J genome came from a DBA/2J origin, including almost all of MMUX. This type of analysis also indicates that at least one additional strain, possibly 129-like, contributed genomic content to C57BLKS/J in regions where the allelic pattern matches neither DBA nor C57BL/6J.

A lack of sufficient underlying SNP data to this point have prevented the thorough development and testing of an algorithm to carry out in silico mapping (Chesler et al. 2001; Darvasi 2001). Previously published methods were severely limited by the lack of SNP density and strain coverage leading to a method that utilized generalized genetic distances and resulted in lack of resolution in the analysis (Grupe et al. 2001; Smith et al. 2003). Based on the data above, this SNP set provides sufficient spacing and resolution to distinguish discrete ancestral patterns, allowing for subsequent in silico analyses to treat the genetic measure used in these calculations as categorical. Although the number of SNPs used here still does not allow the precise definition of haplotype blocks, the relatively even spacing of the SNPs every 300 kb does allow for an inference of ancestral relationships across 1-megabase (Mb) regions. For this reason, a sliding window of three SNPs is used to infer haplotypes at each locus. Strains showing the same pattern are grouped in the same inferred haplotype, as a single category, and any variations are considered to form distinct inferred haplotypes. All of the strain-distribution patterns created by this definition of inferred haplotype were compared across the genome to determine their uniqueness. Replication of the same strain-distribution pattern at multiple locations across the genome, or “mirror loci,” would result in regions that are all equally associated with the phenotype and produce false positives. No occurrences of mirror loci were found outside of a 5-Mb region of any three-SNP block.

**Figure 3:**
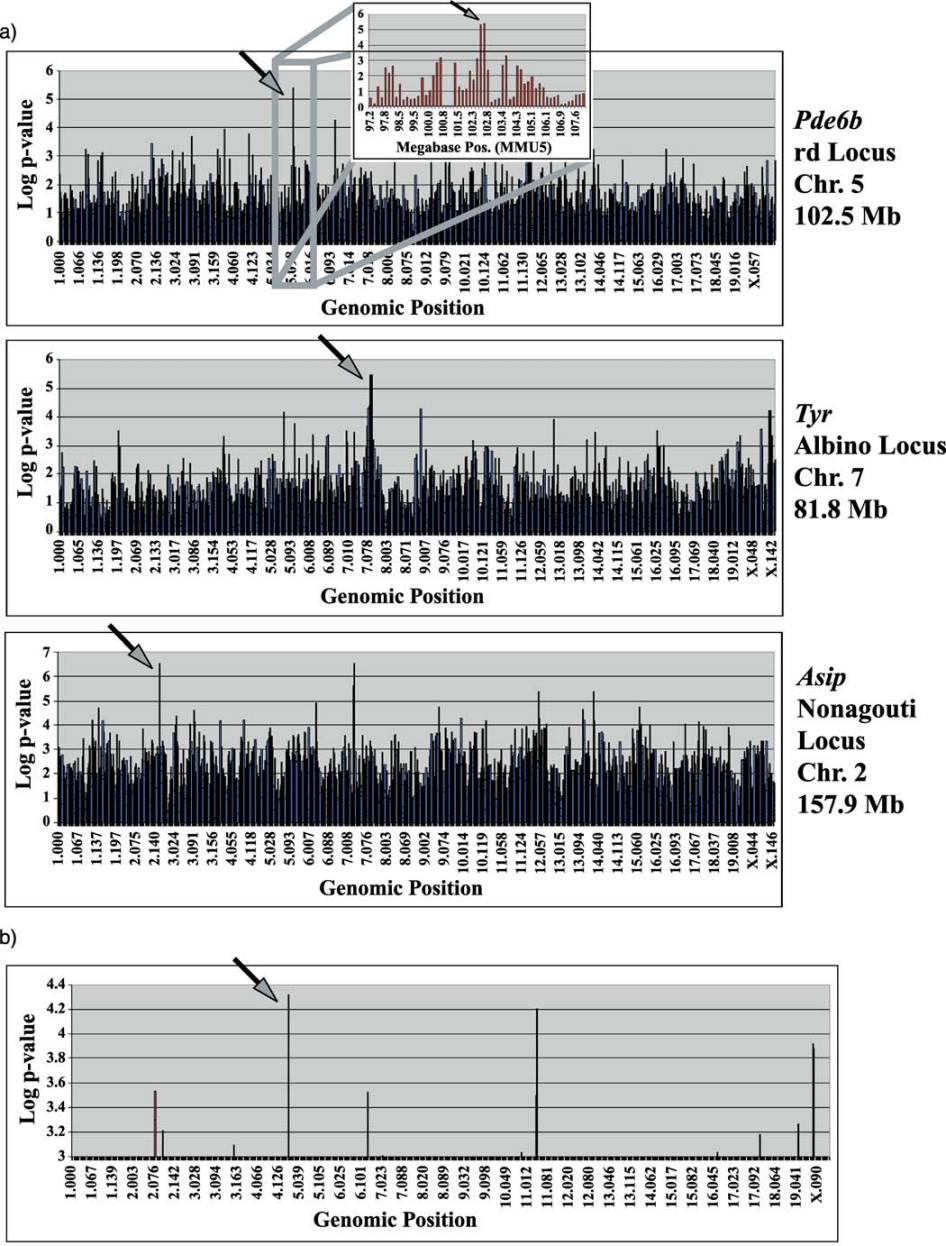
In Silico Mapping Method Correctly Identifies Coat Color, Retinal Degeneration, and Sweet Preference Loci from SNP Data (A) Presence or absence of the retinal degeneration, albino, or agouti phenotypes was given a numerical value of 1 or 0 for use in the mapping algorithm. In each case, the most significant *p*-value (indicated by an arrow) was obtained for the region that contains the gene known to produce these phenotypes. A closer inspection of the retinal degeneration mapping shows that the maximum linkage region indicated by the algorithm covered a 0.4-Mb region from 102.4 Mb to 102.8 Mb on MMU5. (B) *Tas1r3* is known to be a major control gene for the complex trait of preference for sweet tastes. Values for the sweet preference of 23 strains of mice produced a highly significant association with the one Mb region of MMU4 that contains *Tas1r3.*

With this in mind a logistic regression model followed by analysis of deviance was used to determine the association between a sliding window of three SNPs and phenotype scores of 1 or 0 for the presence or absence of three Mendelian traits: coat color traits of nonagouti and albino and retinal degeneration. All of the phenotypes were determined from phenotypic descriptions in The Jackson Laboratory mouse database (http://jaxmice.jax.org/jaxmice-cgi/jaxmicedb.cgi). Albino mice were excluded from the mapping of nonagouti because the nature of the phenotype prevents the ascertainment of agouti or nonagouti coat colors. In each case, the appropriate locus for the gene responsible for the particular trait was identified from this SNP collection with the most significant *p*-value (Figure 3A; Kwon et al. 1987; Bowes et al. 1990; Bultman et al. 1992). Interestingly, for in silico mapping of the nonagouti locus, a highly significant score was also obtained for a locus on MMU7 at 29.9 Mb. This is also the approximate location of the *dark* locus, an unidentified gene that also influences coat color (Silvers 1979).

The ability to properly identify causative genes for monogenic traits is a minimum requirement for a viable in silico mapping method, but to serve its intended purpose it must be able to point to controlling loci when multiple genes act in concert to contribute to a phenotype. To examine a quantitative trait, data from a two-bottle saccharin preference test for 23 strains of mice were analyzed with the Fisher permutation-based analysis of variance (ANOVA) statistical model. Briefly, at each three-SNP window, a modified F-statistic based on the true genotype–phenotype pairings is calculated (detailed in Materials and Methods). The significance of this test statistic is estimated by comparing to a distribution of 1 million random bootstrap samples of phenotypic values. A three-SNP window beginning with marker 04.155.136 obtained the lowest *p*-value of the genome scan (Figure 3B). This locus corresponds to the position of the gene, *Tas1r3,* identified by traditional QTL methods as a primary contributor to the variability of the sweet preference quantitative trait (Bachmanov et al. 2001; Max et al. 2001) Three other saccharin preference QTL were also found to overlap significant associations from this mapping (Table 1).

**Table 1:**
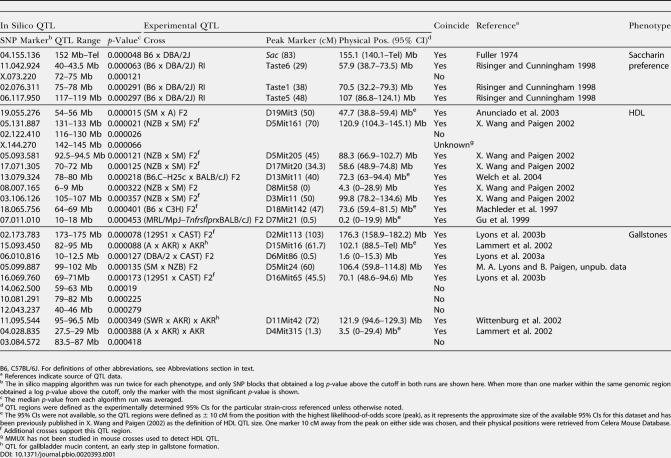
Comparison of In Silico QTL with Experimentally Derived QTL

B6, C57BL/6J. For definitions of other abbreviations, see Abbreviations section in text

^a^ References indicate source of QTL data

^b^ The in silico mapping algorithm was run twice for each phenotype, and only SNP blocks that obtained a log *p*-value above the cutoff in both runs are shown here. When more than one marker within the same genomic region obtained a log *p*-value above the cutoff, only the marker with the most significant *p*-value is shown

^c^ The median *p*-value from each algorithm run was averaged

^d^ QTL regions were defined as the experimentally determined 95% CIs for the particular strain-cross referenced unless otherwise noted

^e^ The 95% CIs were not available, so the QTL regions were defined as ± 10 cM from the position with the highest likelihood-of-odds score (peak), as it represents the approximate size of the available 95% CIs for this dataset and has been previously published in X. Wang and Paigen (2002) as the definition of HDL QTL size. One marker 10 cM away from the peak on either side was chosen, and their physical positions were retrieved from Celera Mouse Database

^f^ Additional crosses support this QTL region

^g^ MMUX has not been studied in mouse crosses used to detect HDL QTL

^h^ QTL for gallbladder mucin content, an early step in gallstone formation

After validation with both monogenic traits and a quantitative trait, the same strategy was applied to map quantitative trait loci for the control of plasma high-density lipoprotein cholesterol (HDL) and gallstone development. The average HDL values from 10-wk-old mice fed on a normal chow diet were taken from the Mouse Phenome Database (Paigen et al. 2002; Bogue 2003). Because of the complexity of these traits, a conservative approach was used for strain selection. Data used for only 23 of the most related laboratory strains and two of the *M. musculus domesticus* strains because if a strain is from a unique lineage and contains unique haplotypes, it will not add any power to the analysis and risks increasing the level of noise (see Materials and Methods for a list of the 25 strains). Using a three-SNP window to analyze the 25 strains, there were no mirror loci present, and on average, 3.8 distinct inferred haplotypes were found at each locus.

Where multiple loci may be expected to be found, as is the case for multigenic traits, a significance threshold was defined. To determine the false positive rate of each *p*-value, a recently described method by Dudoit et al. (2004) was used. The generalized family-wise error rate (gFWER) method uses a bootstrap estimation of the null distribution to assign a significance cutoff. In the case of the HDL phenotype, a significance threshold associated with a false positive rate of less than 0.005 (*p*-value = 0.000506; −log*[p]* = 3.2958) was used (Figure S2). Nineteen three-SNP windows were identified as having significant association with the HDL phenotype, which collapsed into 11 distinct loci (Figure S3). To gauge the reliability of the in silico predictions, the results were compared to previously described QTL regions. Nine of these 11 loci fell within one of the regions identified by traditional two-strain crosses (Table 1). Of the two that were not found to match previously identified QTL, the in silico MMUX QTL would not be expected to be matched because MMUX has been excluded from consideration in prior HDL QTL work.

This same type of analysis was repeated for a phenotype that scored the formation of gallstones in 25 strains of male mice (Paigen et al. 2000). Eleven regions were produced that exceeded the gFWER false positive cutoff (*p*-value = 0.000398; −log*[p]* = 3.400117), and seven of these regions fell within the range of traditionally identified QTL for gallstone formation or mucin accumulation, which is considered a precursor to gallstone formation (Table 1; D. Q. Wang et al. 1997).

As well as identifying QTL, the inferred haplotype data from this SNP set also can be used to assist the narrowing of candidate regions, aiding in the selection of candidate genes. An association for a region overlapping an HDL QTL previously identified on MMU8 did not meet the stringent statistical cutoff set for the in silico method (X. Wang and B. Paigen 2002). The most significant *p*-values obtained for the MMU8 QTL region were consistently found between 89–94 Mb. Sample sequencing of the region confirmed, at a slightly higher resolution, the SNP pattern that generated the association. This sequencing also replicated an inferred haplotype break point in the BTBR strain that narrowed the region to 88.52–90.88 Mb. A candidate gene within this 2-Mb region, *adenylate cyclase 7 (Adcy7),* located at 89.55 Mb, is expressed in the liver and adipose tissue (http://symatlas.gnf.org) and functions by producing cyclic adenosine monophosphate (Watson et al. 1994). Cyclic adenosine monophosphate is known to be an important signaling component in the pathway to lipolysis (Cammisotto and Bukowiecki 2002). Homologous regions containing the rat and human ortholog of *Adcy7* have also been identified as containing an HDL QTL (Bottger et al. 1996; Mahaney et al. 2003; Pajukanta et al. 2003).

**Figure 4:**
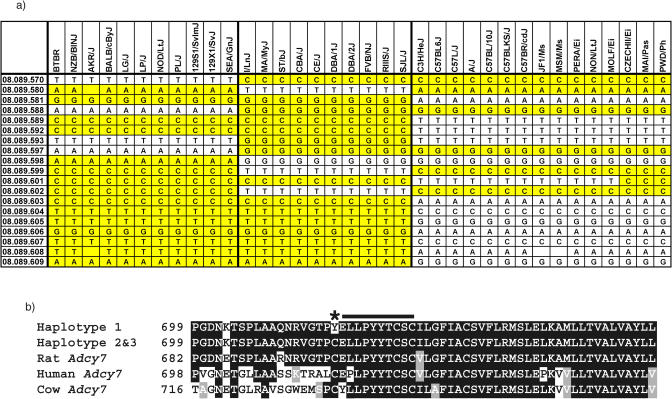
Analysis of *Adcy7* Haplotypes Reveals Amino Acid Change Associated with HDL Phenotype (A) Sequencing of *Adcy7* in multiple strains revealed 28 SNPs distinguishing three distinct haplotype patterns. All strains were typed with markers selected to represent the three haplotypes. The strain distribution pattern predicted by the SNP data and the sample sequencing for this region was confirmed with NZB/BlNJ and BTBR T+ tf/J, I/LnJ and MA/MyJ, and C3H/HeJ, C57BL6/J, and C57L/J, each separating into unique haplotypes. (B) The SNP represented by marker 08.089.597 resulted in a change from a cysteine to a tyrosine in the resulting protein (asterisk). This cysteine is conserved in orthologs of the gene in human, rat, and cow. It is also found at the beginning of a stretch of ten amino acids (indicated by black line) predicted to be one of the protein's ten transmembrane domains. Identical amino acids are black and conserved amino acid changes are gray.

*Adcy7* was sequenced in strains representing the three inferred haplotypes identified for this locus in the SNP dataset. Twenty-eight SNPs were identified in the gene, three of which produced amino acid changes. Nineteen of these SNPs, including the three nonsynonymous changes, were typed against all 48 strains of mice (Figure 4A). One of the haplotypes showed a higher average HDL level than all the others (77.5 mg/dl + 20.3 versus 67.2 mg/dl + 24.3 and 57.9 mg/dl + 16.7). This haplotype also contained a SNP causing a C717Y change in exon 20. Among the 48 strains, the members of this haplotype are the only ones with a replacement of this cysteine, which is conserved in the rat, cow, and human versions of the gene (Figure 4B), making it a good candidate for being a gene that contributes to the variability of HDL levels in the blood (Abiola et al. 2003).

## Discussion

The SNP data here provide new resources for traditional mapping projects and enable development of inbred strain haplotype methods for QTL detection. The analyses presented here indicate that the inferred haplotype structures derived from this dataset provide sufficient estimation of genetic diversity/similarity to map Mendelian traits to within 1-Mb intervals. QTL can also be defined as inferred haplotype loci of several megabases in size. The analysis for QTL provides a rank order of significant phenotype/genotype associations, and using the gFWER method of controlling for multiple-testing error, the loci reported as statistically significant are very likely to be biologically relevant. This point is borne out by the high concordance between the in silico QTL and the traditionally determined QTL (Table 1). The traditionally determined HDL QTL identified in the mouse covers 42% of the genome and are in concordance with nine of ten in silico QTL—a significant result (*p* < 0.0025). This excludes the MMUX in silico QTL since they cannot be verified from current traditional QTL data. The false positive cutoff employed here is very restrictive and could be relaxed to find additional real associations, but the chances of including false positives would then increase. For the gallstones phenotype the concordance is not demonstrated to the same level; however, the top-ranked loci still show overlap with previously defined QTL. What about the loci that do not show overlap; are they still real? From a statistical analysis it is unlikely they are false positives. In these results, 25 strains are simultaneously combined, unlike standard QTL mapping using two-strain comparisons, and some phenotype–genotype associations may occur that have not been observed by classical methods. Contributions from diverse strains that have not normally been used in F2 crosses or available in RI lines can now be incorporated.

Even showing that this method does find significant associations, the question arises about its general utility and applicability. The methods of in silico mapping as described here should be viewed as a complement to, and not a substitute for, traditional methods for mapping QTL. Although we have demonstrated a robust approach to in silico mapping, it would certainly not be expected to find all QTL for a given phenotype. Major contributors to phenotypic variation will show up, but weaker contributors would be expected to be lost because of the limited power of 25 strains. It would also be expected to miss QTL resulting from recent strain-specific mutations or low-frequency haplotypes. Traditional QTL methods will still be required to identify the more subtle interactions, including those involving epistasis and modifying genes.

However, these methods would provide a useful starting point for a new phenotype that is being investigated, where often the first step for any QTL analysis is a strain survey to quantify the range of the phenotype. Additionally, if these analyses are overlaid with the results from a traditional two-strain QTL mapping, one of the major advantages to be gained from this approach is that associative loci are defined in terms of a few megabases instead of tens of centiMorgans.

The number and selection of strains and appropriate phenotype are also important considerations. Here we have limited our analysis of the complex traits, HDL and gallstones, to 25 strains—those that are best interrogated by this SNP set. While it is true that more strains have the potential to add greater statistical power to resolve QTL, this potential is limited by our ability to accurately represent the ancestral relationship of those additional strains. If we add more strains, but cannot accurately infer haplotype structures in those strains, we only add more noise to the analysis. The ability to detect all possible haplotypes in the utilized strains from the SNP data suffers from the availability of sequence data, currently from only four strains of mice. Because the source SNPs come from the sequencing of only four closely related strains, this current set is biased toward interrogating ancestry of *M. m. domesticus.* To be successful, phenotypes must have a low intrastrain variation but sufficient variance within the strain set selected. This however, is not a requirement restricted to in silico mapping.

The overall power of this method will only improve as the biases and limitations of the SNP panel are addressed and additional strains are genotyped and phenotyped. Unique strains would become more useful if all possible SNPs are known and the mapping is then done directly with the causative polymorphism or at least with a large unbiased set of SNPs. As resequencing of other mouse genomes progresses, the ability to correctly infer the complete number and structure of haplotypes will improve, and the number of QTL regions reaching statistically significant levels will increase.

Recently, two similar studies of haplotype structures across 5-Mb regions were published, although they produced differing conclusions on how their findings might affect in silico mapping efforts (Frazer et al. 2004; Yalcin et al. 2004). Yalcin et al. has suggested that the complex nature of mouse haplotype structure and the small size of many haplotypes in inbred strains will make in silico mapping methods untenable and will preclude the mapping of any meaningful genotype–phenotype association short of whole genome resequencing (Yalcin et al. 2004). This assessment would presumably hold true even for the well-defined Mendelian traits. The inferred haplotypes from a three-SNP window spanning on average 900 kb would not be able to reflect ancestral relationships, so the appropriate genotype–phenotype association could not be made no matter the strength of the allele in determining phenotype. Yet, clearly they can. The Frazer et al. (2004) study, which utilized more strains and produced significantly greater coverage of their 5-Mb region, estimates that the average ancestral segment length among classical inbred strains is in the order of 1.5 Mb in size, within the resolution of this work. In fact, the Yalcin et al. (2004) data show similar megabase-long ancestral relationships between strain pairs (for example, 5 Mb of near identity between A/J and C3H/J). This in silico approach concurs with the conclusions of Frazer et al. (2004). Despite the complexities of haplotype structures, the use of a large enough set of strains with a dense SNP map does allow for significant and real associations to be found.

This is not to suggest that fragmented small haplotypes are not common in the genome of the inbred mouse. This clearly does mean that there will be regions of the genome that will not be interrogated well by an in silico method. This approach is still limited by the density of this SNP map and can only be expected to visualize inferred haplotype patterns of approximately 1 Mb in size, and therefore smaller haplotype structures are hidden and potential phenotype–genotype associations will be missed. Here future, larger SNP sets that will allow more SNPs to infer haplotype will become important. However, this is the best resolved whole genome view of the diversity of the commonly used inbred strains to date.

The algorithms employed here provide a starting point for further development of in silico mapping. We have shown that they can be used to identify Mendelian traits and replicate classical QTL associations. Clearly, the next goals are to validate some of the previously unreported associations, and this work is ongoing.

## Materials and Methods

### 

#### SNP selection and detection

SNPs for use in genotyping were selected on a weighted basis from the Celera Mouse SNP Database containing data from the DBA/2J, A/J, C57BL/6J, 129S1/SvImJ, and 129X1/SvJ strains. Sufficient SNPs were selected for coverage of at least one SNP per 300 kb on average. The 129S1/SvImJ and 129X1/SvJ strains were considered as the same strain when their alleles agreed; preference was given first to SNPs where each allele of the SNP was represented by two strains. This was done to favor selection of SNPs representing ancestral inheritance, not recent strain-specific mutations, and to favor real SNPs as opposed to errors in sequence annotation. Additional selective value was incorporated based on whether the SNP was in a gene, how many sequencing runs supported the presence of the SNP, and the proximity of the SNP to previously selected SNPs. Additional SNPs used to characterize the *Tas1r3* locus were gathered from sequence from multiple strains available in GenBank (http://www.ncbi.nlm.nih.gov/entrez/query.fcgi?db=nucleotide&cmd=search&term=tas1r3). All physical positions presented in the paper are from the Celera Mouse Genome Assembly R13.

Primers for PCR and single-base extension were designed by using the SpectroDESIGNER software package (Sequenom, San Diego, California, United States). Assay designs are available as Supporting Information. All SNP assays were named for their position in the genome in the following format: the chromosomal location, the Mb position on the chromosome, and the kb position with a period separating each number.

For SNP genotyping, genomic DNA from pedigreed mice (Mouse DNA Resources, The Jackson Laboratory, Bar Harbor, Maine, United States) was diluted to 10 ng/μl, and 1 μl of DNA was combined with 2.45 μl of water, 0.1μl of 25 mM dNTPs (Invitrogen, Carlsbad, California, United States), 0.03μl of 5 units/μl HotStar *Taq* (Qiagen, Valencia, California, United States), 0.625 μl of 10X HotStar PCR buffer containing 15 mM MgCl_2_, 0.5μl PCR primers mixed together at a concentration of 1.25 μM for multiplexed reactions, and 0.325 μl of 25 mM MgCl_2_. Reactions were heated at 95 °C for 15 min followed by 45 cycles at 95 °C for 20 s, 56 °C for 30 s, and 72 °C for 1 min and a final incubation at 72 °C for 3 min. After PCR amplification, remaining dNTPs were dephosphorylated by adding 1.5 μl of water, 0.17 μl of homogeneous mass extend reaction buffer (Sequenom), 0.3 units of shrimp alkaline phosphatase (Sequenom), and 0.03 μl of 10 units/μl exonuclease (USB Corporation, Cleveland, Ohio, United States). The reaction was placed at 37 °C for 20 min, and the enzyme was deactivated by incubating at 85 °C for 15 min. After shrimp alkaline phosphatase treatment, the genotyping reaction was combined with 0.76 μl of water, 0.2 μl of 10X Termination mix (Sequenom), 0.04 μl of 0.063 units/μl Thermosequenase (Sequenom), and 1μl of 10 mM extension primer. The MassEXTEND reaction was carried out at 94 °C for 2 min and then 99 cycles of 94 °C for 5 s, 52 °C for 5 s, and 72 °C for 5 s The reaction mix was desalted by adding 3 mg of a cationic resin, SpectroCLEAN (Sequenom), and resuspended in 30 μl of water. Completed genotyping reactions were spotted in nanoliter volumes onto a matrix arrayed into 384 elements on a silicon chip (Sequenom SpectroCHIP), and the allele-specific mass of the extension product was determined by matrix-assisted laser desorption ionization time-of-flight MS. Analysis of data was by automated allele calling from the SpectroTYPER software. All SNP data are available at NCBI dbSNP (http://www.ncbi.nih.gov/entrez/query.fcgi?db=snp) and The Jackson Laboratory Mouse Phenome Database (http://www.jax.org/phenome). Placement of the SNP data across the genome and major and minor allele distributions can be visualized using SNPview (http://snp.gnf.org).

#### Statistical modeling for in silico mapping

The use of a single marker is restrictive in the sense that it only allows a representation of the genome as diallelic. The use of windows of multiple markers enables the visualization of more complex genomic relationships between multiple strains. This more accurately models actual haplotype patterns than does a binary approach. In determining the size of the SNP window to use as a definition of inferred haplotype for purposes of the algorithm, sizes of two, three, four, and five SNPs were examined. A window of only two SNPs was still found to be too limiting. Windows of three, four, and five SNPs produced similar results, but as window size is increased biologically meaningful patterns become fragmented, creating more single-strain inferred haplotypes, resulting in an increase in noise. Singly represented haplotypes can never be informative in this analysis because the commonality of haplotypes is required to achieve significant association with a phenotype. Three SNP windows were also analyzed across the whole genome to identify mirror loci. This would be a locus that has exactly the same strain distribution pattern across all 25 strains used in an in silico run. There were no mirror loci, or 1-off, or 2-off mirror loci (with one or two strains not grouped identically) that occurred outside of a 5-Mb interval.

Defining the genetic measure as a categorical unit necessitated the use of an ANOVA-based model. The type of ANOVA to use was determined by the characteristics of the phenotypic values.

The phenotypes studied here fell into two categories: binary or continuous. The coat color phenotype was considered as binary, where phenotypic values were set to 1 and 0. The HDL phenotype is an example of a continuous phenotype since the phenotypic values are measured on a continuous scale. Two different statistical methods were employed based on this distinction.

When phenotypic values are binary, the appropriate statistical approach involves first fitting a binomial generalized linear model to the data. An analysis of deviance table is then computed for the fitted model. The R language function *glm* with the parameter *family* set to *binomial* was used. This was followed by an application of *anova.glm* with the parameter *test* set to *Chisq.*

For continuous phenotypic values, a log transformation was applied to reduce the effects of outliers in the phenotypic data. Next, an F-statistic weighted for the genotypic diversity of the strains within an inferred haplotype group was used. The weighted F-statistic had the following form:



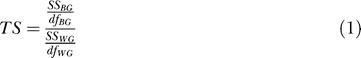



where



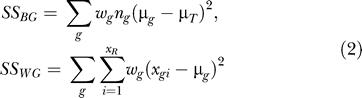



and



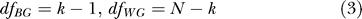



where *n_g_* is the number of strains in a given inferred haplotype, *μ_g_* is the mean of phenotypic values in a given inferred haplotype , *μ_T_* is the mean of all phenotypic values, *k* is the number of inferred haplotypes, *N* is the total number of data values, and *w_g_* is the weight representing the genetic diversity of the inferred haplotype. The genetic diversity ratio (*w_g_*) between two strains is the number of SNPs genome-wide in which both strains have genetic information and they disagree, divided by the total number of SNPs in which they both have genetic information. The genetic diversity coefficient for an inferred haplotype in the weighted F-statistic is the average *w_g_* between all strain pairs contained in the inferred haplotype.

The weighted F-statistic calculated at each SNP window determines if at least one of the inferred haplotypes has an average phenotypic value significantly different from the other inferred haplotypes. To assess the significance of the computed value, the null distribution of the weighted F-statistic was simulated at each SNP window by taking a million bootstrap samples of the phenotypic values. As in the algorithm used for binary phenotypes, inferred haplotype patterns present in only one strain were not included in the calculation because they are not informative in elucidating shared ancestral blocks. From this distribution of a million random F-statistics, 200 bootstrap samples of size 1 million were computed. For each bootstrap sample, a *p*-value was computed by dividing the number of random F-statistics larger than the true F-statistic by the total number of random F-statistics (million). In this way 200 *p*-values were collected. The vertical heights reported in the bar graphs (see Figure S2) are the −log(*p*) transform of the median of these 200 *p*-values. A 95% confidence interval (CI) for the *p*-value at this window was also calculated from this bootstrap distribution.

To estimate the overall false positive rate for this type of calculation, calculating a significance threshold based on the family-wise error rate (FWER) has been proposed (Churchill and Doerge 1994). Others have noted that the traditional FWER calculation is too strict in the context of multiple testing and leads to a significant loss of power (Lander and Kruglyak 1995). Therefore, we employed a recently developed method of bootstrap estimation of common cutoffs based on the gFWER (Dudoit et al. 2004). Whereas the FWER method reports significance, using the most conservative criterion of only one false positive, the gFWER method controls for multiple testing while allowing for an acceptable false positive rate (in our case, α < 0.005).

The gFWER method to control for false positives as applied to in silico mapping is briefly described as follows. A null reference distribution was constructed using random bootstrap tests to determine a significance cutoff. Ten thousand bootstrap samples of phenotype values were randomly assigned to the true haplotype structure. For each random bootstrap sample, the nonparametric ANOVA approach outlined above was performed at each three-SNP window, with one difference. Whereas the initial true calculation reports the median of 200 bootstrap *p*-values, the gFWER method requires an estimate of the “supremum” (least upper bound) of expected values reported at each locus, so the most significant value is reported from the 200 bootstrap *p*-values (following Procedure 3 in Dudoit et al. 2004), ensuring a conservative false positive estimate. For each bootstrap sample, the genome-wide −log(*p*-value) corresponding to the (1 − α) percentile was added to the null distribution (as described in Procedure 5, Dudoit et al. 2004). Finally, after the 10,000 bootstraps are complete, the significance threshold is set as the (1 −α) percentile in the entire null reference distribution (computed from our 10,000 randomly bootstrapped iterations). While this threshold still represents a conservative estimate of the desired false positive rate, the gFWER has significantly more power than the traditional FWER calculation.

Using this method for calculation of false positives, it is not necessary to specify the marginal distribution of the test statistic at each window of SNPs. Estimations of false positives or power that assume some parametric form of test statistic's distribution are not reliable in this context. This distribution can alter radically at each SNP window. In this context, the statistical problem of calculating quantities like discovery power (that is, ultimately the type I and type II error) is further complicated. Nearly 11,000 hypothesis tests (one at each three-SNP window) are conducted in a single run of the algorithm. Therefore, equations that currently exist for the estimation of power for QTL mapping by traditional methods cannot be applied here because they assume that the test statistic has some previously defined parametric form. Code for the above described algorithms is available upon request.

For calculation of the significance of the number of in silico QTL that overlapped with previously identified QTL for the HDL phenotype, a binomial distribution was used



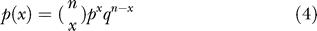



given *p* = probability of success of 0.42 (overlap with HDL QTL in previous literature).

Therefore *q* = probability of failure; the 0.0025 result is the probability of at least nine successes in ten trials. Only ten loci could be assessed for this result as no information is available for traditional HDL QTL present on the X chromosome.

For the mapping of the retinal degeneration traits, 37 strains were used. This represented all of the strains for which information existed in The Jackson Laboratory database minus the most divergent wild-derived strains for which inference of haplotype would be expected to be most inaccurate. These strains were A/J, AKR/J, BALB/cByJ, BUB/BnJ, C3H/HeJ, C57BL/10J, C57BL/6J, C57BLKS/J, C57BR/cdJ, C57 l/J, C58/J, CBA/J, CE/J, DBA/1J, DBA/2J, FVB/NJ, I/LnJ, KK/HlJ, LG/J, LP/J, MA/MyJ, NOD/LtJ, NON/LtJ, NZB/BlNJ, NZW/LacJ, PERA/EiJ, PL/J, RIIIS/J, SEA/GnJ, SJL/J, SM/J, ST/bJ, SWR/J, WSB/EiJ, ZALENDE/EiJ, 129S1/SvImJ, and 129X1/SvJ. Because of the added complexity of the coat color traits, mapping was restricted to the 25 most related strains for which coat color phenotype could clearly be determined. For the albino analysis, 129S1/SvImJ, A/J, AKR/J, BALB/cByJ, C3H/HeJ, C57BL/10J, C57BL/6J, C57BLKS/J, C57BR/cdJ, C57 l/J, C58/J, CBA/J, DBA/1J, DBA/2J, I/LnJ, LP/J, MA/MyJ, NZB/BlNJ, NZW/LacJ, PERA/EiJ, PL/J, SEA/GnJ, SM/J, WSB/EiJ, and ZALENDE/EiJ strains were used. For the nonagouti mapping, the same strain set as the albino mapping was used except for the mice presenting the albino phenotype. The strains were 129S1/SvImJ, C3H/HeJ, C57BL/10J, C57BL/6J, C57BLKS/J, C57BR/cdJ, C57 l/J, C58/J, CBA/J, DBA/1J, DBA/2J, I/LnJ, LP/J, NZB/BlNJ, PERA/EiJ, SEA/GnJ, SM/J, WSB/EiJ, and ZALENDE/EiJ. Any mouse showing an agouti coat color was considered to be agouti for this analysis regardless of genotype at the agouti locus. Only limited phenotype data were available for saccharin preference, so again all strains with available data except the most divergent wild-derived strains for which inference of haplotype would be expected to be most inaccurate were used. These strains were A/J, AKR/J, BALB/cByJ, BUB/BnJ, C3H/HeJ, C57BL/6J, C57 L/J, CBA/J, CE/J, DBA/2J, FVB/NJ, I/LnJ, KK/HlJ, LP/J, NOD/LtJ, NZB/BlNJ, PL/J, RIIIS/J, SEA/GnJ, SJL/J, SM/J, ST/bJ, and SWR/J. For the mapping of the other complex traits, only the 25 strains with the closest ancestral relationship were used. These strains were 129S1/SvImJ, A/J, AKR/J, BALB/cByJ, BTBR T+ tf/J, C3H/HeJ, C57BL/10J, C57BL/6J, C57BLKS/J, C57BR/cdJ, C57 l/J, C58/J, CBA/J, DBA/1J, DBA/2J, I/LnJ, LP/J, MA/MyJ, NZB/BlNJ, NZW/LacJ, PERA/EiJ, PL/J, SEA/GnJ, SM/J, and WSB/EiJ.

## Supporting Information

Dataset S1 Complete Allele Call and Assay List (16.1 MB XLS).

Figure S1 Phylogenic Tree of 48 Strains Generated from SNP Dataset Ancestral relationships between strains can be seen within clusters of the tree such as the fact that BALB/cByJ is a progenitor strain to SEA/GnJ. The bias of the SNP set can also be viewed by the exaggerated distance between the C57 and 129 clusters and the DBA and A/J cluster. The wild-derived strains make up the outermost cluster, but the three *M. m. domesticus* strains show a much closer relationship than the other wild-derived strains to the common laboratory strains.
(2.2 MB EPS).

Figure S2 Duplicate In Silico Genome Scans for the HDL Phenotype The log *p*-value at each three-SNP window was calculated and plotted along the x-axis. Because any log *p*-value below 3 will not reach significance, calculations are halted at any locus once obtaining a log *p*-value of 3 becomes impossible in order to increase computational throughput. As such all log *p*-values below 3 are reported at 3. The false positive cutoff established by the gFWER calculation is indicated by a horizontal red line. Every quantitative trait was run twice through the algorithm to ensure consistency of results.
(5.8 MB EPS).

Figure S3 Distribution of log *p-*Values from gFWER Calculation of Significance for HDL In Silico Analysis To estimate an appropriate false positive cutoff, 10,000 genome scans are conducted on randomized datasets and the 99.5 percentile log *p*-value is reported from each run. The significance cutoff is indicated by the vertical red line.
(3.1 MB EPS).

Table S1 Frequency of Polymorphic Alleles between Strain Pairs (44 KB XLS).

Table S2 Total Number of SNP Alleles between Strain Pairs (34 KB XLS).

### Accession Numbers

The Mouse Phenome Database (http://www.jax.org/phenome) accession numbers for the phenomes discussed in this paper are MPD:29 and MPD:99.

## Abbreviations

*Adcy7*: *adenylate cyclase 7*
ANOVA: analysis of variance
CI: confidence interval
gFWER: generalized family-wise error rate
HDL: high-density lipoprotein cholesterol
Mb: megabase
MMU: Mus musculus chromosome
QTL: quantitative trait loci
RI: recombinant inbred
kb: kilobase
SNP: single nucleotide polymorphism
